# Genome Wide Association Study of Fetal Hemoglobin in Sickle Cell Anemia in Tanzania

**DOI:** 10.1371/journal.pone.0111464

**Published:** 2014-11-05

**Authors:** Siana Nkya Mtatiro, Tarjinder Singh, Helen Rooks, Josephine Mgaya, Harvest Mariki, Deogratius Soka, Bruno Mmbando, Evarist Msaki, Iris Kolder, Swee Lay Thein, Stephan Menzel, Sharon E. Cox, Julie Makani, Jeffrey C. Barrett

**Affiliations:** 1 Muhimbili Wellcome Programme, Department of Haematology and Blood Transfusion, Muhimbili University of Health and Allied Science, Dar-es-salaam, Tanzania; 2 Department of Biological Sciences, Dar es Salaam University College of Education, Dar-es-salaam, Tanzania; 3 Department of Human genetics, Wellcome Trust Sanger Institute, Cambridge, United Kingdom; 4 King’s College London, Department of Molecular Haematology, Division of Cancer Studies, London, United Kingdom; 5 National Institute for Medical Research, Tanga Centre, Tanga, Tanzania; 6 King’s College Hospital NHS Foundation Trust, Department of Haematological Medicine, London, United Kingdom; 7 MRC International Nutrition Group, Faculty of Epidemiology & Population Health, London School of Hygiene & Tropical Medicine, London, United Kingdom; 8 Nuffield Department of Medicine, University of Oxford, Oxford, United Kingdom; University of North Carolina, United States of America

## Abstract

**Background:**

Fetal hemoglobin (HbF) is an important modulator of sickle cell disease (SCD). HbF has previously been shown to be affected by variants at three loci on chromosomes 2, 6 and 11, but it is likely that additional loci remain to be discovered.

**Methods and Findings:**

We conducted a genome-wide association study (GWAS) in 1,213 SCA (HbSS/HbSβ^0^) patients in Tanzania. Genotyping was done with Illumina Omni2.5 array and imputation using 1000 Genomes Phase I release data. Association with HbF was analysed using a linear mixed model to control for complex population structure within our study. We successfully replicated known associations for HbF near *BCL11A* and the *HBS1L*-*MYB* intergenic polymorphisms (HMIP), including multiple independent effects near *BCL11A*, consistent with previous reports. We observed eight additional associations with P<10^−6^. These associations could not be replicated in a SCA population in the UK.

**Conclusions:**

This is the largest GWAS study in SCA in Africa. We have confirmed known associations and identified new genetic associations with HbF that require further replication in SCA populations in Africa.

## Introduction

Tanzania has one of the highest number of births of individuals with sickle cell disease (SCD) in the world, estimated to be between 8,000 and 11,000 births a year [1]. Fetal hemoglobin (HbF) is a major ameliorating factor in SCD. Patients with higher HbF levels have less pain [2], [3], lower morbidity and improved survival [2], [4]. The inter-individual HbF variation has been associated with variants at three principal loci [5]–[7]; the region on chromosome 11p that contains the *HBB* itself [8] and loci on chromosomes 2p (*BCL11A*) and 6q (*HBS1L-MYB* intergenic polymorphism, *HMIP*). Rare variants in *KLF1* have also been reported to influence HbF levels [9]. Of the three principal loci influencing HbF, *BCL11A* variants have been found to be more prevalent in the British, American and Brazilian patients of African descent [6]–[8], [10], [11] than *HMIP* and *HBB* variants. Similarly, in Tanzania *BCL11A* variants (*rs11886868* and *rs4671393*) had the highest overall impact, with *rs4671393* alone accounting for up to 12.8% HbF variance [12]. Notably, most of the reported *BCL11A* variants reside within the intron 2 of the gene [6], , and appear to occur in moderate to high linkage disequilibrium (LD) in African ancestry [15] in Southwest USA (ASW) [17]. This suggests that African populations may be suitable for the identification and fine mapping of the causal variant/s for the *BCL11A* locus. Together, the 3 loci have been reported to account for up to 50% of HbF variation in non-anemic individuals in Europe [13]. However, a large proportion of HbF variance in African populations remains unaccounted for, that justifies the need for genetic studies of HbF in Africa.

We performed a genome wide association study (GWAS) in 1,213 individuals with SCA in Tanzania to identify new genetic loci associated with HbF levels and any additional variants of the known 3 loci.

## Materials and Methods

### Study population: Discovery and Replication

**Figure 1:**
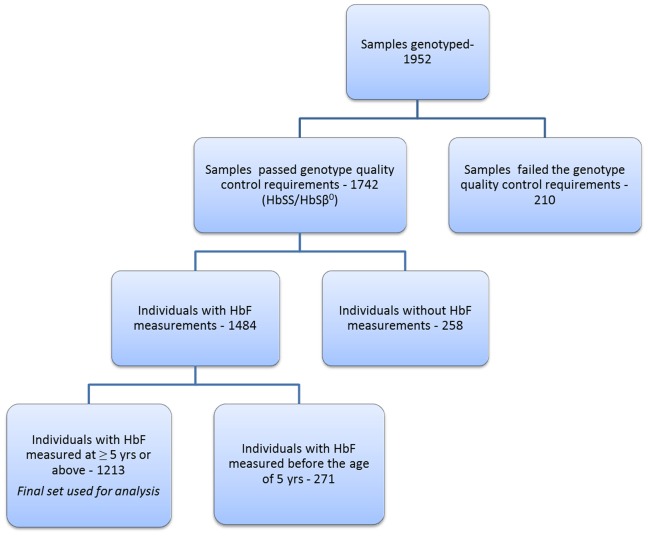
A flow chart diagram showing the flow of samples at different stages of the study. The genetic quality control measures involved removal of individuals with abnormal heterozygosity, gender mismatch, missingness of data, removal of duplicate samples and low quality/rare variants.

1952 samples were collected and genotyped from individuals who had a diagnosis of SCD. These individuals are part of the Muhimbili Sickle Cohort recruited at Muhimbili National Hospital, Dar es Salaam, Tanzania. At Muhimbili, HbF% measurements are performed by High Performance Liquid Chromatography (HPLC) (Variant I Hemoglobin Testing System, Biorad, Hercules, CA) on all individuals with SCA HbF data used in this study was measured at steady state (defined as malaria test negative, no reported current pain, fever, blood transfusion or hospitalization within 30 days (before or after the date of blood collection). Patients on hydroxyurea and those suspected to be HbS/β+ were excluded from the study. Figure 1 shows the flow of samples at different stages of this study. 1742 SCA (HbSS/HbSβ^0^) individuals had successful genotype data (see quality control section below) among which 1484 individuals had HbF values collected. For this study we used HbF data collected at five years of age and above. Therefore, a total of 1213 individuals with successful genotype (only HbSS and HbSβ^0^) and HbF data were included in the discovery cohort. Written informed consent was obtained from patients/guardians and ethical approval was obtained from the Muhimbili University Research and publications committee (MU/RP/AEC/VOL.XIII). The replication cohort included 321 patients (HbSS and HbS/β^0^) enrolled through the Hematology Outpatient Clinic in King’s College Hospital, London, with written informed consent obtained for all patients. Ethical approval was granted by the King’s College Hospital, London, Local Research Ethics Committee, protocol no 01–083.

### Genotyping and replication

DNA was extracted from archived buffy coat samples from patients collected at enrollment using Nucleon BACC II genomic DNA extraction kits from GE Healthcare life sciences. Samples were typed on the Illumina Human Omnichip 2.5 platform (Illumina Inc., San Diego,CA, USA) which assays ∼2.4M Single Nucleotide Polymorphisms (SNPs). For the replication, 16 SNPs from 10 loci were selected and assayed using TaqMan (Applied Biosystems, Foster City, Ca).

### Statistical analysis

#### Quality Control

**Figure 2:**
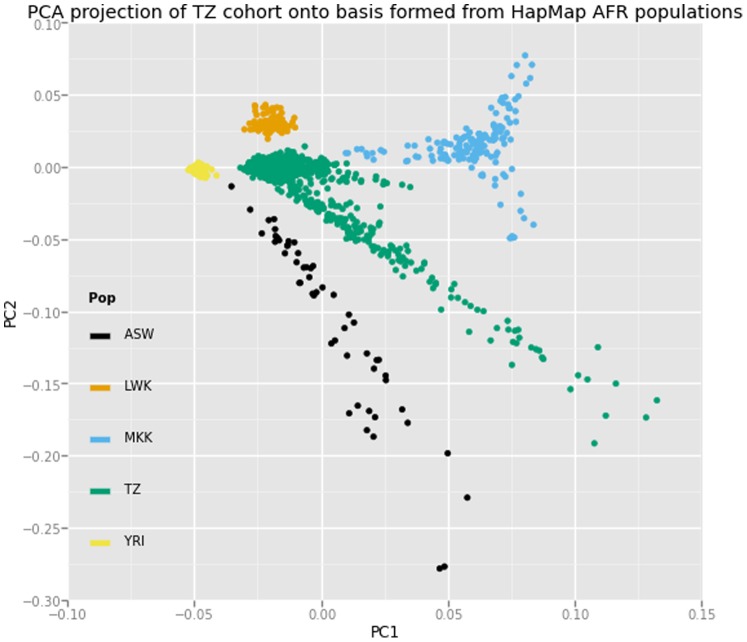
Principal components plot (PC2 v PC1) with bases formed using genotypes of individuals of African descent included in Hapmap3 study. ASW: African ancestry in Southwest USA, LWK: Luhya in Webuhe, Kenya, MKK: Maasai in Kinyawa, Kenya, TZ: Tanzanian study population, YRI: Yoruba in Ibadan, Nigeria.

Standard technical QC was performed using PLINK to remove potential sources of technical and genetic bias [18]. A total of 85 samples either found with abnormal heterozygosity (defined as 3 standard deviation from the mean), gender mismatch, and those with more than 3% of missing data were excluded from the study. Following identity-by-descent analysis, we removed duplicate samples and identified individuals in first, second, and third-degree relationships. Principal components analysis (PCA) was performed using EIGENSTRAT with 1000 Genomes Phase 1 populations as reference groups [19]. We observed population substructure and admixture within the cohort (Figure 2), and identified 262 individuals showing clear separation from the core population. Following sample removal, rare and low quality variants (more than 3% data missing,<10^−6^ Hardy-Weinberg chi-square P-values, and <1% MAF) were excluded, resulting in a typed dataset of 1,827,523 variants in 1,742 individuals.

Imputation was performed using IMPUTE 2.0 with the 1000 Genomes Phase I integrated variant set release as the primary reference panel [20]. Low quality imputed variants were removed (<0.4 INFO score and <1% MAF), with 15,153,765 SNPs and indels remained for association testing.

### HbF association analysis

We carried out our genome-wide association analysis on the 1213 individuals described above. The distribution of HbF levels was first standardized using three methods: a log transformation, a power transformation with a lambda of 1/3, and empirical normal quantile transformation. The transformation methods yielded consistent results in the later association analyses. In order to take into account population stratification, genetic and cryptic relatedness, we used a linear mixed model framework as implemented in the program MMM [21]. We adjusted for sex and age as fixed covariates, and allowed for a random effect with variance dependent on the genetic relatedness between individuals as determined by a genome-wide panel of SNPs. LD-based clumping was performed using PLINK to identify independent signals amongst variants with suggestive significance (P<10^−5^). To calculate the variance explained by our top loci, we performed additive regressions in R (http://www.r-projects.org/) using threshold-called genotypes after controlling for sex, age, and 10 principal components. Conditional analyses were performed using MMM with each conditioned variant included as a fixed effect covariate. We generated quantile-quantile (Q-Q) and Manhattan plots using R, and regional association plots using LocusZoom [22].

## Results

**Figure 3:**
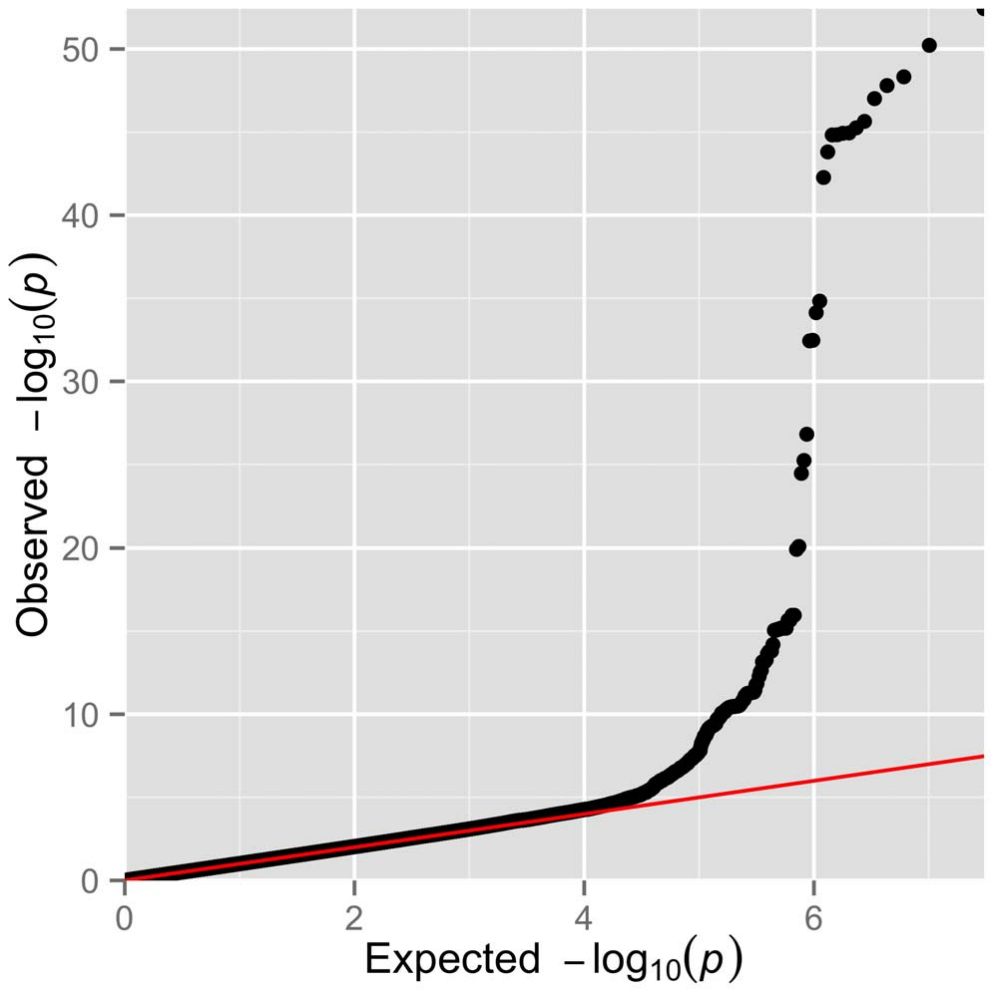
Summary of the genome-wide association results of normalized HbF within Tanzanian individuals with SCA. Q–Q plot of the observed versus the expected P-values from a linear mixed model for the entire set of 15,153,765 SNPs.

**Figure 4:**
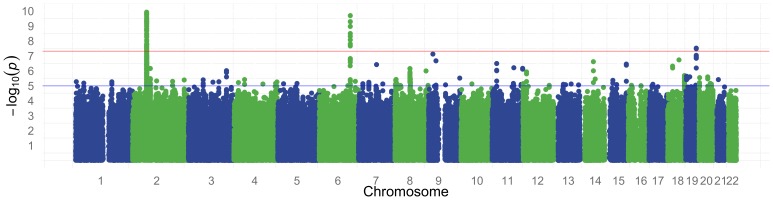
Manhattan plot for normalized HbF association results (-log_10_P) plotted against the position on each chromosome. The red horizontal line indicates genome wide suggestive at 5×10^−8^ and blue line indicates genome wide suggestive at 1×10^−5^.

We performed a genome wide association study for HbF in a discovery cohort of 1,213 patients (52.6% females) with SCA and a replication cohort of 321 patients (54.8% females) of African Caribbean descent or West African descent. Details on age, sex and HbF levels are presented in Table 1. The genomic control (λ_GC_) for the analysed SNPs was 1.0156 and a Q–Q plot of the observed versus expected P-values is shown in Figure 3. The absence of an early departure of the observed P-values suggests that our data are not affected by problems with genotyping, imputation, and uncontrolled sample relatedness or population stratification. The distribution of association P-values (Manhattan plot) for HbF level is shown in Figure 4.

**Table 1:** Characteristics of SCA populations in Tanzania and UK.

	Tanzania	UK
Number	1,213	321
Gender (% female)	52.6	54.8
Age, Y	11 [IQR: 7.9–16.5]	25 [IQR:19–36]
HbF%	4.6 [IQR: 2.5–7.7]	6 [IQR: 2.9–10.3]

As in previous genome wide studies, we have strongly replicated 2 loci that have been associated with HbF: near *BCL11A* (*rs1427407*, Beta = −0.30, P = 3.74×10^−53^) on chromosome 2 and the *HMIP* (*rs9494145*, Beta = 0.23, P = 2.42×10^−10^) situated on chromosome 6. Notably, none of the variants situated at the *HBB* and *KLF* loci could be replicated in our study. We have detected 8 novel loci with genome wide significance of P<1×10^−6^ (Table 2). Of these, the top 3 significant SNPs include; *rs28567737* (Beta = −0.12, P = 4.26×10^−8^) situated on chromosome 19, *rs10468869* (Beta = 0.09, P = 1.33×10^−7^) on chromosome 18 and *rs62573842* (Beta = 0.11, P = 1.88×10^−7^) on chromosome 9. However, none of the novel loci could be validated in our replication set (Table 2).

**Table 2:** SNPs associated with HbF levels in individuals with SCA in Tanzania and UK.

			Discovery Cohort (n = 1213) Replication Cohort (n = 321)							
Chr	SNP	Position	Ref/alt allele	Gene	EAF	β-value	P value	EAF	B-value	P-value
2	*rs1427407*	60718043	T/G	*BCL11A*	0.78	−0.30	3.74×10^−53^	0.55	−0.36	0.003
6	*rs9494145*	135432552	T/C	*HBS1L-MYB*	0.05	0.23	2.42×10^10^	0.01	0.33	0.049
7	*rs6466533*	78792892	T/C	*MAG12*	0.24	0.10	3.59×10	0.03	0.07	0.47
9	*rs10756993*	18839724	A/C	*ADAMTSL1*	0.95	−0.27	2.01×10	–	–	–
9	*rs62573842*	32264312	A/G		0.20	0.11	1.88×10	0.02	0.02	0.79
11	*rs6590706*	133334906	G/A	*OPCML*	0.80	0.11	2.32×10	0.61	0.08	0.34
14	*rs192197462*	57582783	G/A		0.47	−0.10	2.34×10	–	–	–
15	*rs7163278*	93888391	T/C	*AK094352*	0.09	0.17	4.72×10	0.02	0.09	0.38
18	*rs10468869*	49321773	G/A		0.59	0.09	1.33×10	0.48	0.03	0.68
19	*rs28567737*	46531219	T/C	*CCDC61/MIR769/*	0.21 *PGLYRPI*	−0.12	4.26×10	0.08	0.07	0.69

Abbreviations: Chr: chromosome, SNP: single nucleotide polymorphism, ref allele: ancestral allele, alt allele: alternative allele, EAF: effect allele frequency, β-value (Beta estimate): the effect that a change in single allele has on the trait,-β indicate a decreasing effect. Chromosomal position (position) given here uses hg19 co-ordinates.

The SNP with the strongest signal (*rs1427407*, Beta = −0.30, P = 3.74×10^−53^) was situated in the second intron of *BCL11A*. Our initial analysis highlighted the presence of several SNPs in LD (R^2^) with *rs1427407* (our top signal). To gain further insight on this region, we performed a stepwise conditional regression analysis (Table 3). After conditioning on *rs1427407*, there was a significant increase in association at *rs6545816* (P = 2.55×10^−15^ compared to P = 0.58 before conditioning). This finding suggests an independent *BCL11A* effect that might be tagged by *rs6545816.* A third conditional analysis using the first two *BCL11A* variants identified a third candidate variant, *rs58955256* with suggestive significance (P = 1.52×10^−6^).

**Table 3:** Association summary of genome wide significant *BCL11A* SNPs from non-conditional and conditional regression analysis.

							Non-conditional		conditional	
							*rs1427407*		*rs6545816*	
Chr	SNP	BP	ref allele	alt allele	β-value	P-value	β-value	P-value	β-value	P-value
2	*rs58955256*	60583070	A	G	−0.10	6.95×10	−0.11	1.07×10	−0.09	1.52×10
2	*rs6545816*	60714861	A	C	0.01	0.58	0.14	2.55×10^−15^	–	–
2	*rs1427407*	60718043	T	G	−0.30	3.74×10^−53^	–	–	–	–

Abbreviations: Chr: chromosome, SNP: single nucleotide polymorphism, ref allele: ancestral allele, alt allele: alternative allele, β-value (Beta estimate): the effect that a change in single allele has on HbF,-β indicate a decreasing effect. Chromosomal position (BP) given here uses hg19 co-ordinates.

## Discussion

GWAS have been previously applied in the identification of genetic variants that regulate levels of HbF, both in healthy individuals and those with SCA [5]–[8], [12]. To date, three primary loci; *BCL11A*, *HMIP* and *HBB* at chromosomes 2, 6 and 11 have been reported to account for 20%–50% of HbF inter-individual variation in non-anemic Europeans [13]. However, the contribution of these loci in African populations has been estimated to be lower [8]. Our study represents the first GWAS in 1213 SCA patients from a single site in Africa.

**Figure 5:**
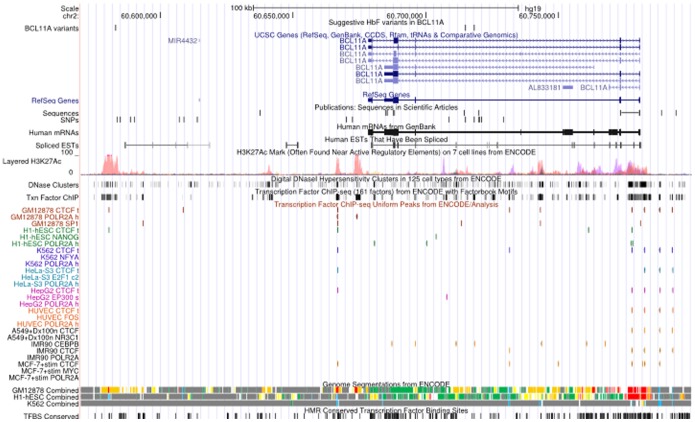
A snapshot of UCSC genome browser showing relevant ENCODE tracks. The positions of variants reported in this study have been indicated within a track labelled as “suggestive HbF variants in *BCL11A*”. The positions of variants reported in previous studies.

Our findings have confirmed the genetic association of *BCL11A* and *HMIP* in the regulation of HbF. Our work also supports the likely causality of *rs1427407* at the *BCL11A* locus, as recently reported [16], as well as the presence of multiple independent risk variants. The *rs1427407* falls within a peak of GATA1 andTAL1 binding and the minor T allele is believed to disrupt a composite motif bound by GATA1 [16]. *Rs6545816* sits 3kb from *rs1427407* while *rs58955256* is further upstream of *rs1427407* (130 kb). These variants sit near regions with regulative activity within the BCL11A gene (Figure 5), however, their specific functions have not yet been reported and should be considered for further research.

Although we validated the *HMIP* association, the allele frequency was extremely low (6%) resulting in a much smaller amount of HbF variance explained by the locus (2.54%) compared to other populations. It is worth noting that this SNP resides within block 2 of *HMIP*, a region where most of the significant SNPs from other studies are located. The *HBB* association with HbF was below the genome wide significance level with most variants with P values of x10^−5^reason for this could be that *HBB* variants are rare in our population, as reflected in allele frequencies (EAF of less than 5%) reported in our previous study [12] In a study in Cameroon, the *HBB* variant (*rs7482144*) studied was also found to be rare (MAF = 0.07) [23]. With a better sample size such as in meta-analyses, it may be possible to improve the association signal. Similarly, the *KLF1* association with HbF that has been previously reported [9] was not detected in this study.

**Figure 6:**
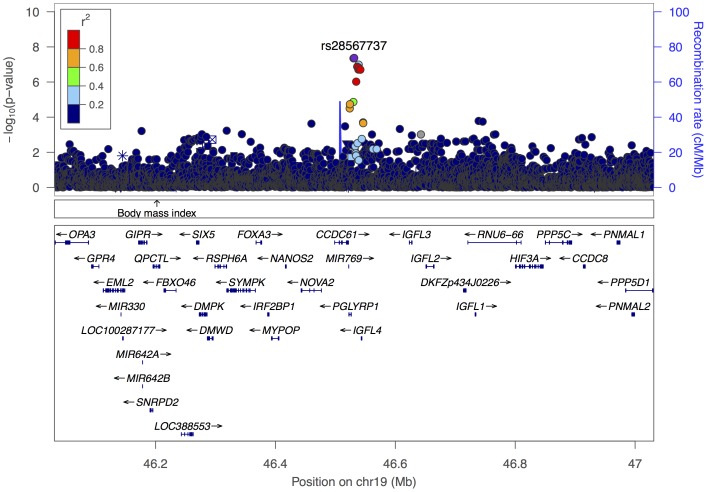
Regional association plot of the chromosome 19 region with our suggestive novel SNP (*rs28567737*). The left y axis represents the negative log10 P values of the associations of SNPs in this region. Each SNP is represented by a dot, with grey dots indicating low LD with *rs28567737*. The right y axis represents the recombination rate (in centimorgans [cM] per megabase [Mb]). Genes found in the region are shown in relative position under the plot; arrows indicate the direction of transcription.

The discovery of additional loci has become increasingly challenging mostly because the effect size of a new locus is likely to be lower than that of the 3 primary loci. Our study has identified 8 suggestive novel SNPs worthy of follow up in larger studies. One of these, *rs28567737* on chromosome 19 (Figure 6), was genome-wide significant (4.9×10^−8^), and was in high LD (0.991) with *rs10414361* (P = 1.31×10^−7^), a variant directly typed on the Omni2.5 assay. The lack of validation of the suggestive loci in the UK cohort may be due to the small sample size or different ancestry. The latter is likely to be more significant as most of the UK patients have a Caribbean and West African origin while the population in our study is from East Africa. In addition, studies of SCD suggest that there is a considerable heterogeneity in the genetic and environmental composition of SCD populations within Africa [23], [24] let alone in different continents. Based on differences in ancestry, it is likely that these populations would have different sickle haplotypes with the majority of individuals within the UK cohort carrying the Senegal/Benin haplotype while most of the Tanzanian patients would carry the Bantu/Central African Republic (CAR) in. In addition, HbF regulation pathways may develop differently in different populations [8], hence, allele frequencies may differ across populations. Such a difference has been previously observed for *HMIP* tag SNP (*rs9399137*) variant which was found to be less common in the Tanzanian (MAF = 0.01) compared with the UK patients (MAF = 0.07) [12]. In this study we observed lower minor allele frequencies for the suggestive variants within the replication cohort compared to the discovery cohort (Table 2). Another difference observed was high levels of HbF in the UK cohort than those of Tanzanian patients. It is therefore critical that replication studies are done with cohorts within the same geographical region as it is likely that there will be more homogeneity in ancestry. The best replication population for our study would have been from East Africa, however, SCA studies with required HbF data and DNA samples are scarce.

This study highlights the importance of studying disease relevant phenotypes in large populations from individual sites in Africa in order to characterize genetic risk with locale-specific allele frequencies. We believe datasets such as this will form the foundation of international meta-analyses of SCA and other diseases prevalent in Africa.
